# The potential link between Covid‐19 and multiple myeloma: A new saga

**DOI:** 10.1002/iid3.701

**Published:** 2022-11-18

**Authors:** Hayder M. Al‐Kuraishy, Ali I. Al‐Gareeb, Ali A Mohammed, Athanasios Alexiou, Marios Papadakis, Gaber El‐Saber Batiha

**Affiliations:** ^1^ Department of Clinical Pharmacology and Medicine, College of Medicine ALmustansiriyia University Baghdad Iraq; ^2^ The Chest Clinic, Barts Health NHS Trust Whipps Cross University Hospital London UK; ^3^ Department of Science and Engineering Novel Global Community Educational Foundation Hebersham Australia; ^4^ AFNP Med Wien Austria; ^5^ Department of Surgery II, University Hospital Witten‐Herdecke University of Witten‐Herdecke Wuppertal Germany; ^6^ Department of Pharmacology and Therapeutics, Faculty of Veterinary Medicine Damanhour University Damanhour Egypt

**Keywords:** acute lung injury, acute respiratory distress syndrome, Covid‐19, multiple myeloma, SARS‐CoV‐2

## Abstract

**Background:**

Covid‐19 is considered a primary respiratory disease‐causing viral pneumonia and, in severe cases, leads to acute lung injury and acute respiratory distress syndrome (ARDS). In addition, though, extra‐pulmonary manifestations of Covid‐19 have been shown. Furthermore, severe acute respiratory distress syndrome coronavirus type 2 (SARS‐CoV‐2) infection may coexist with several malignancies, including multiple myeloma (MM).

**Methods:**

This critical literature review aimed to find the potential association between SARS‐CoV‐2 infection and MM in Covid‐19 patients with underlying MM. Narrative literature and databases search revealed that ARDS is developed in both MM and Covid‐19 due to hypercalcemia and proteasome dysfunction.

**Results:**

Notably, the expression of angiogenic factors and glutamine deficiency could link Covid‐19 severity and MM in the pathogenesis of cardiovascular complications. MM and Covid‐19 share thrombosis as a typical complication; unlike thrombosis in Covid‐19, which reflects disease severity, thrombosis does not reflect disease severity in MM. In both conditions, thromboprophylaxis is essential to prevent pulmonary thrombosis and other thromboembolic disorders. Moreover, Covid‐19 may exacerbate the development of acute kidney injury and neurological complications in MM patients.

**Conclusion:**

These findings highlighted that MM patients might be a risk group for Covid‐19 severity due to underlying immunosuppression and most of those patients need specific management in the Covid‐19 era.

## Graphical abstract

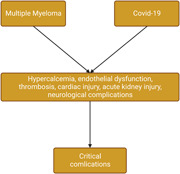

## 1 INTRODUCTION

A novel severe acute respiratory distress syndrome coronavirus type 2 (SARS‐CoV‐2) has been established as the reason for a global pandemic coronavirus disease 2019 (Covid‐19).^1^ Covid‐19 is considered a primary respiratory disease‐causing viral pneumonia and, in severe cases, leads to acute lung injury (ALI) and acute respiratory distress syndrome (ARDS).^2^ In addition, extrapulmonary manifestations of Covid‐19, including neurological, cardiovascular, gastrointestinal, renal, and metabolic complications, have been shown.^3^ The clinical presentation of Covid‐19 is asymptomatic or mild in most cases (85%).^4^ However, about 15% of Covid‐19 patients may be presented with a severe form due to the development of ALI that requires hospitalization.^5^ Remarkably, 3%−5% of severe Covid‐19 patients may progress to a critical stage that requires mechanical ventilation and intensive care admission due to ARDS progression.^6^

The pathogenesis of SARS‐CoV‐2 infection occurs by binding this virus to the angiotensin‐converting enzyme 2 (ACE2), which is highly expressed in various cell types, including immune cells.^7^ A direct cytopathic effect of SARS‐CoV‐2 infection activates immunological and inflammatory responses, which are resolved after viral clearance.^7^ Nevertheless, exaggerated immune response and release of a high amount of proinflammatory cytokines may sometimes advance, causing hyper inflammation and cytokine storm.^8^ Consequently, direct, and indirect effects of SARS‐CoV‐2 infection may lead to systemic effects and the development of multiorgan injury (MOI).^8^

SARS‐CoV‐2 infection may coexist with several malignancies, including multiple myeloma (MM). The rationale of the present study was that hyperinflammation, Multiple organ failure, and thrombosis in MM might be augmented during SARS‐CoV‐2 infection, causing more complications. Therefore, the present critical review aimed to find the potential association between SARS‐CoV‐2 infection and MM in Covid‐19 patients with underlying MM.^9^

## 2 MM

MM or plasma cell myeloma is the malignant proliferation of plasma cells.^9^ The causes of MM are unknown; however, obesity, radiation, and some chemicals may increase the risk for the development of MM.^10^ Monoclonal gammopathy may develop into smoldering myeloma, which is plasma cell dyscrasia characterized by high monoclonal paraprotein. Smoldering myeloma may progress to plasma cell leukemia or MM.^11^ The proliferation of plasma cells in MM may form a mass in the soft tissue and bone marrow known as plasmacytoma.^12^ Patients with MM are asymptomatic initially. Then with the progress of the disease, it presented with fatigue, bone pain, and recurrent infections due to the development of anemia, kidney dysfunction, and immunosuppression.^13^ MM is more common in men around the age of 60 years, with a prevalence of 0.7%. Without treatment, the median survival of patients with MM is approximately 7 months. Treatment survival is 4−5 years, and 5‐year survival is 54%.^14^

The diagnostic criteria of MM including; hypercalcemia (>11 mg/dl), renal insufficiency (serum creatine >2 mg/dl), anemia (Hb < 10 g/dl), osteolytic lesions, clonal plasma cells >10% on bone marrow biopsy and myeloma protein >3 g/dl in plasma or urine.^15^ Bone pain affects 70% of patients with MM, usually affects ribs and spines, and causes pathological fractures.^16^ The underlying cause of bone pain in MM is activating the receptor activator for nuclear factor kappa B ligand (RANKL), which activates osteoclasts in bone marrow stroma resulting in resorptive lesions and calcium release.^16^ Increased intraosseous pressures and bone marrow acidity could also cause bone pain in patients with MM.^16^ Anemia in MM results from suppression of bone marrow for the production of erythrocytes by inflammatory cytokines or infiltration of bone marrow by malignant plasma cells.^17^

Moreover, hyper/hypogammaglobulinemia, hypercalcemia, and amyloidosis are the most common causes of the development of renal insufficiency in MM.^18^ Also, immune deficiency in MM increases the risk for secondary bacterial infections.^19^

### 2.1 Pathogenesis of MM

MM is a monoclonal tumor of antibody‐secreting plasma cells in the bone marrow that is often diagnosed by the presence of a typical M‐spike by serum protein electrophoresis or by free light chains in the urine. Its symptomatic phase is associated with significant end‐organ damage, including lytic bone lesions, anemia, loss of kidney function, immunodeficiency, and amyloid deposits in various tissues.^20^ MM cells are the malignant counterparts of post‐germinal center long‐lived plasma cells characterized by strong bone marrow dependence, somatic hypermutation of immunoglobulin genes, and isotype class switch resulting in the absence of IgM expression in all but 1% of tumors.^21^ However, MM cells differ from healthy plasma cells because they retain the potential for a low proliferation rate. Virtually every case of MM is preceded by a premalignant plasma cells tumor called monoclonal gammopathy of undetermined significance^20^, ^21^, ^22^ that, like MM, produces a typical M‐spike by a free light chain in the urine. It has to be distinguished from an IgM‐secreting lymphoid, a precursor phase of chronic lymphocytic leukemia, lymphoplasmacytoma, and Waldenstrom's macroglobulinemia.^20^, ^21^, ^22^, ^23^, ^24^ Plasma cells tumor is age dependent in about 4% of individuals over 50^21^ and can progress to MM at average rates of 1% per year. Monoclonal gammopathy of undetermined significance is distinguished from MM by having an M‐spike of <30 g/L, with no more than 10% of bone marrow mononuclear cells being tumor cells and no end organ damage or other symptoms. Progression of monoclonal gammopathy of undetermined significance to smoldering MM and symptomatic MM is associated with an expanding bone marrow tumor mass and increasingly severe organ impairment or symptoms.^1^ Despite the recent advances in understanding MM pathogenesis, it is still largely impossible to predict which monoclonal gammopathy of undetermined significance patient will and which one will never progress to MM. Although MM cells are characterized by a strong dependence on the bone marrow tumor microenvironment, at the late stages of the disease, the more aggressive tumor may sometimes extend to extramedullary locations, such as the spleen, liver, and extracellular spaces. Extramedullary MM can also present with a leukemic phase classified as secondary or primary plasma cell leukemia, depending on whether or not a preceding intramedullary MM was recognized.^20^, ^21^, ^22^, ^23^, ^24^ Most of the available human MM cell lines have been generated from extramedullary MM and represent a renewable repository of the oncogenic events involved in the initiation and progression of the most aggressive end‐stage MM tumors.^24^

In MM, overproducing proinflammatory cytokines, mainly Interleukin‐6 (IL‐6), leads to osteoporosis and angiogenesis.^21^ Similarly, uncontrolled production of antibodies and their deposition in different organs may cause MOI and complications like renal failure and polyneuropathy.^23^

Moreover, in MM, there is uncontrolled proliferation of plasma cells due to the translocation of promoter genes to the chromosome, resulting in the activation of the antibody gene to produce more antibodies.^22^ A chromosomal translocation between genes of immunoglobulin heavy chain on chromosome 14 and oncogene is commonly observed in MM.^20^ This translocation induces uncontrolled activation of oncogenes with consequent clonal proliferation of plasma cells. Abnormalities in both chromosomes 13 and 14 are observed in 50% of MM.^20^

Progressive genetic and epigenetic disorders in the bone marrow plasma cells provoke malignant proliferation of these cells with further monoclonal proliferation of plasma cells in various tissues to form plasmacytoma or progress to develop plasma cell leukemia. It has been reported that 2%−4% of MM progress to plasma cell leukemia.^24^

## 3 THE LINK BETWEEN COVID‐19 AND MM

### 3.1 ARDS

ARDS is the main fatal complication of severe Covid‐19.^25^ In severe SARS‐CoV‐2 infection, the release of damage‐associated molecular patterns (DAMPs) from infected cells stimulates toll‐like receptors 4 (TLR4) with subsequent release of proinflammatory.^25^ These changes trigger the development of pulmonary infiltration, lung edema, endothelial inflammation, abnormal immune activation, and vascular thrombosis resulting in the impairment of alveolar homeostasis and the development of ARDS.^26^

Different studies illustrated that MM might cause ARDS due to lung involvement by neoplastic plasma cells due to diffuse alveolar infiltration.^27^ However, ARDS had been reported in MM without pulmonary parenchymal infiltration, which might be due to osteolytic rib changes and the development of the flail chest.^27^ Diaphragmatic paralysis due to phrenic nerve mononeuropathy may cause respiratory paralysis and hypercapnia.^28^ Ravinet et al. reported a case of 61 years old man with chronic emphysema who developed MM and then respiratory failure. Postmortem analysis showed bilateral diffuse nodular infiltration of the lungs by plasma cells, indicating ARDS.^29^ These observations indicated that extramedullary manifestations of MM in the lung might lead to ARDS progression. The underlying mechanism of ARDS in MM is not fully understood; however, hypercalcemia, a common complication in MM, may deposit in the alveolar basement membrane with subsequent induction of inflammation and capillary permeability.^30^ In addition, proteasome dysfunction in the alveolar type 2 epithelial cells increases the risk for ARDS development due to a reduction in the alveolar epithelial barrier function and formation of surfactants.^31^ Proteasome dysfunction is highly evident in MM,^32^ which could potentially cause ARDS development in MM.

In Covid‐19, hypercalcemia is a biomarker linked with poor prognosis and clinical outcomes.^33^ Hypercalcemia in SARS‐CoV‐2 infection is developed due to dysregulation of calcium channels by SARS‐CoV‐2 and associated inflammatory cytokines.^33^ As well, proteasome activation is associated with the development of cytokine storm in Covid‐19.^34^ Therefore, proteasome inhibitors could be effective against cytokine storm in Covid‐19.^35^

These findings suggest that ARDS is developed in both MM and Covid‐19, possibly due to hypercalcemia and proteasome dysfunction.

### 3.2 Cardiovascular complications

Covid‐19 may cause various cardiovascular complications, including endothelial dysfunction, coagulopathy, acute cardiac injury, and blood pressure disturbances.^36^ These pathological changes are developed due to the direct cytopathic effect of SARS‐CoV‐2 infection mediated by the high expression of ACE2 in the cardiovascular system.^37^ Besides, hyperinflammation, cytokine storm, and disturbance in the renin‐angiotensin system in SARS‐CoV‐2 infection may cause severe cardiovascular instability leading to develop heart failure, acute coronary syndrome, and ischemic events.^36^, ^37^

In MM, deposition of amyloid protein and direct toxicity of chemotherapeutic/cytotoxic agents may cause cardiac dysfunction, cardiac conductive disturbances/arrhythmias, and thromboembolic disorders.^38^ Lee et al. found that the use of immunomodulatory and proteasome inhibitors in managing MM may be associated with the development of cardiotoxicity/cardiomyopathy, accelerated hypertension, heart failure, arrhythmia, and coagulopathy.^39^ Of note, carfilzomib, a novel proteasome inhibitor used in MM, is associated with cardiotoxicity. The risk of carfilzomib‐induced cardiotoxicity can be reduced by increasing infusion dose time.^40^

The fundamental causes of cardiovascular complications, mainly heart failure in MM, are angiogenesis, glutaminolysis, and hematological disturbances.^41^ Neoplastic plasma cells and other bone marrow cells have the ability to secrete angiogenic growth factors like vascular endothelial growth factor (VEGF) and fibroblast growth factor 2 (FGF‐2), leading to angiogenesis.^42^ Hypoxia‐inducible factor (HIF) is also released from bone marrow cells due to microenvironment hypoxia.^43^ Elevating HIF in MM is regarded as a compensatory mechanism to protect from tissue hypoxia. HIF controls oxygen homeostasis by regulating the ischemic heart's redox homeostasis and glucose metabolism.^44^ Depleting glutamine in MM due to abnormal metabolism by plasma cells may increase the risk of developing heart failure in MM.^41^ It has been reported that glutamine has a cardioprotective effect against tissue hypoxia.^45^ Also, developing severe anemia in MM may increase the risk of heart failure progression.^46^

In Covid‐19, VEGF and FGF‐2 are upregulated in SARS‐CoV‐2 infection and associated with neuroinflammation advancement.^47^ Hypoxia in Covid‐19 due to the development of ARDS triggers the release of HIF‐1, which in turn increases the expression of VEGF. Further, VEGF induces lung inflammation by increasing capillary permeability and plasma extravasation.^48^ Therefore, VEGF inhibitors like bevacizumab could be of therapeutic benefit in managing ARDS in Covid‐19.^48^ Indeed, HIF‐1 is upregulated in severe Covid‐19 due to hypoxia, and could be a compensatory mechanism to protect tissue against hypoxia.^49^ Thus, HIF‐1 stabilizers may prevent Covid‐19 complications and poor clinical outcomes.^49^

There are different isoforms of HIF‐1; HIF‐1β is stable; its expression is independent of hypoxia and has a long half‐life and anti‐inflammatory properties.^50^ On the contrary, HIF‐1α is an unstable form, activated by hypoxia, has a short half‐life due to metabolism by propyl hydroxylase, and has proinflammatory properties.^51^ Furthermore, HIF‐1α stimulates immune cells to produce and release proinflammatory cytokines and progress cytokine storm; however, HIF‐2α has the opposite effect.^52^ Therefore, inhibitors of HIF‐1α or activators of HIF‐2α might benefit in treating Covid‐19.

On the other hand, glutamine deficiency in different metabolic disorders increases the risk of Covid‐19.^53^ Mohajeri et al. found in a case‐controlled study that glutamine supplementation in Covid‐19 reduced the level of proinflammatory cytokines and associated complications.^54^

Notably, expression of angiogenic factors, HIF‐1, and glutamine deficiency could link Covid‐19 severity and MM in the pathogenesis of cardiovascular complications.

### 3.3 Thrombosis

Outstandingly, thrombosis and other coagulation disorders are regarded as hallmarks in the pathogenesis of SARS‐CoV‐2 infection.^55^ High proinflammatory cytokines in Covid‐19 can interact with platelets and induce the expression of tissue factors and plasminogen activator inhibitor‐1 (PAI‐1).^55^ With suppression of the fibrinolytic pathway and endothelial dysfunction, SARS‐CoV‐2 infection can trigger thrombogenesis.^56^ As well, hypoxia in severe Covid‐19 through induction release of HIF‐1 can induce expression of PAI‐1, adhesion molecules, tissue factor pathway inhibitor, and other procoagulant factors.^55^, ^56^

Thrombotic events and coagulation disorders are also present in patients with MM, mostly those treated with lenalidomide or thalidomide with dexamethasone or anthracyclines.^57^ About one‐third of patients with MM develop thrombosis if not taken thromboprophylaxis.^57^ Of interest, unlike patients with solid tumors in whom coagulation is a marker of poor outcome and prognosis, thrombosis does not affect overall survival in patients with MM.^57^ Surprisingly, molecular mechanisms connecting thrombosis and solid tumors, such as upregulation of PAI‐1, cyclooxygenase‐2 (COX‐2), and tissue factors, are not noteworthy in MM‐mediated thrombosis.^58^ Thus, thrombosis in MM is mainly caused by the therapeutic regimen used to manage this disease.^57^ Therefore, thromboprophylaxis like antiplatelets and anticoagulants, should be initiated with immunotherapy in MM.^59^

Both MM and Covid‐19 share thrombosis as the most typical complication in this state. However, unlike thrombosis in Covid‐19, which reflects disease severity, thrombosis does not reflect disease severity in MM. In both conditions, thromboprophylaxis is essential to prevent pulmonary thrombosis and other thromboembolic disorders.^60^, ^61^

### 3.4 Acute kidney injury (AKI)

AKI is common in Covid‐19 in about 25% due to direct SARS‐CoV‐2 invasion, hemodynamic instability, cytokine storm, endothelial dysfunction, and thrombotic disorders.^62^ These pathological changes trigger acute tubular necrosis, glomerular injury, and collapsing glomerulopathy.^63^, ^64^

In MM, cast nephropathy and tubule‐interstitial injury are the most common causes of AKI in patients with MM. Similarly, increasing the free light chain proteins in MM may lead to tubulointerstitial nephritis and acute tubular necrosis resulting in AKI.^65^ Bridoux and colleagues illustrated that precipitation of uromodulin and monoclonal free light chain proteins in the distal renal tubules leads to AKI in MM.^66^

Notably, thrombosis, endothelial dysfunction, and exaggeration of inflammation state are linked with AKI development in both MM and Covid‐19.^55^, ^57^ Therefore, Covid‐19 may exacerbate and increase AKI risk in patients with MM.

### 3.5 Neurological manifestations

In Covid‐19, different neurological disorders are developed due to direct invasion of the central nervous system (CNS) by SARS‐CoV‐2 or through induction of neuroinflammation by proinflammatory cytokines, which can cross the impaired blood brain barrier.^67^ Dewanjee et al. found that Covid‐19 patients may present with different neurological manifestations due to the involvement of the CNS and peripheral nervous system (PNS).^68^ SARS‐CoV‐2 infection‐causing cerebral vasculitis, endothelial dysfunction, thrombosis, and microcirculatory failure and leads to stroke progression, meningitis, encephalitis, and myelitis.^68^ It has been shown that 36.4% of Covid‐19 patients presented with various neurological manifestations, including headache, ataxia, seizure, stroke, neuropsychiatric disorders, and anosmia.^69^

In MM, CNS involvement is rare, with an incidence of 1% of patients with MM.^70^ Plasma cell infiltration into CNS occurs through the hematogenous route or direct invasion from skull plasmacytoma.^71^ MM's neurological manifestations include headache, ataxia, seizure, and stroke due to a cranial space‐occupying lesion. Likewise, spinal cord injury and radiculopathy due to pathological spine fracture may be developed and could be the presenting symptoms of MM.^72^, ^73^

**Figure 1:**
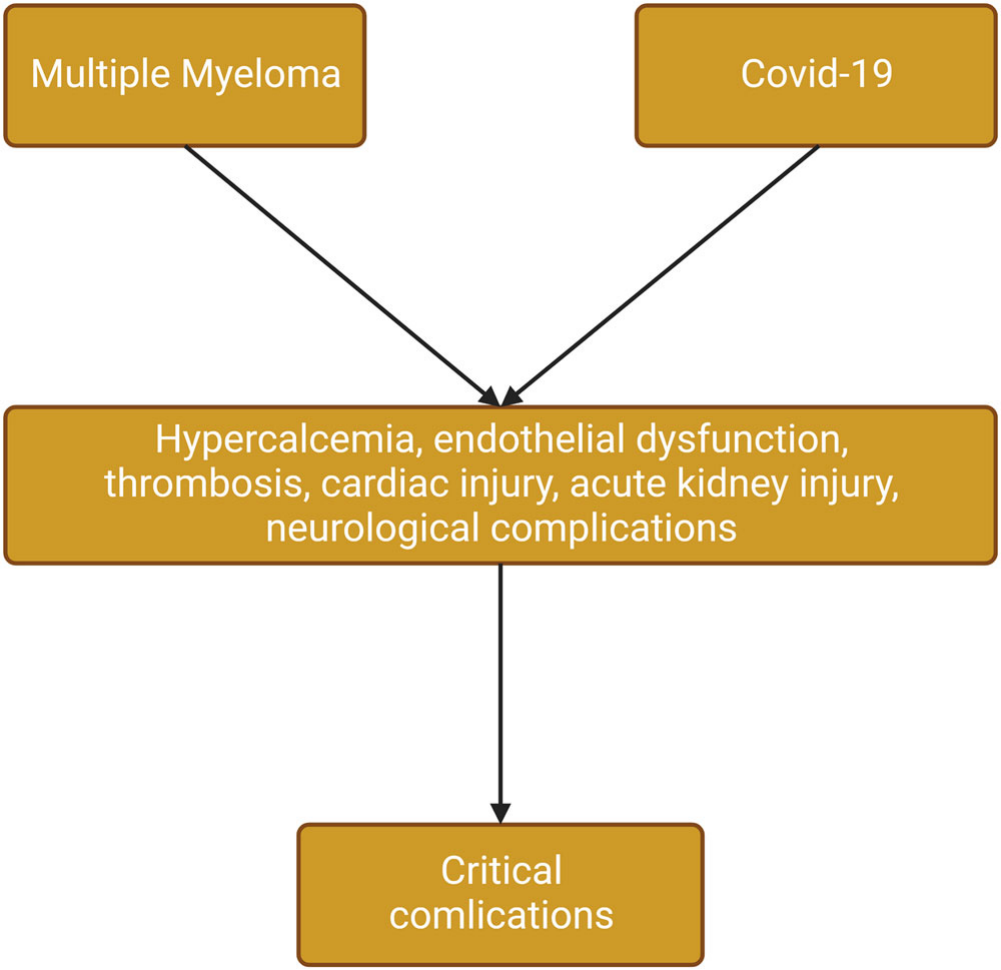
Critical complications in multiple myeloma and Covid‐19

These verdicts suggest that neurological manifestations are more common in Covid‐19 than that in MM. Therefore, the development of Covid‐19 in patients with MM may precipitate neurological complications and increase the risk of poor outcomes. Taken together, the development of Covid‐19 in patients with MM leads to severe complications due to shared endothelial dysfunction, thrombosis, hyperinflammation, acute cardiac injury, acute kidney injury, and neuroinflammation (Figure 1).

## 4 INFLAMMATORY SIGNALING PATHWAYS IN COVID‐19 AND MM

### 4.1 Nod‐like receptor pyrin 3 (NLRP3) inflammasome

Pathogenesis of Covid‐19 and MM is linked with the activation of different signaling pathways, which trigger different inflammatory complications. One of these inflammatory signaling is the NLRP3 inflammasome, highly activated in Covid‐19, resulting in cytokine storm and tissue injury.^74^ NLRP3 inflammasome activates cleavage of caspase‐1 and release of DAMPs and proinflammatory cytokines.^74^ In MM, the accumulation of β2 microglobulin is ingested by MM‐associated macrophages. Uptake and deposition of β2 microglobulin trigger lysosomal rupture and activation of the NLRP3 inflammasome, which through activation of caspase‐1 induces the release of proinflammatory cytokines.^75^ The aberrant expression of the NLRP3 inflammasome/caspase‐1 axis is involved in the pathogenesis of MM and was observed in patients with MM.^76^ Therefore, inhibition of NLRP3 inflammasome may reduce the onset and severity of MM^75^ and can mitigate hyper inflammation in Covid‐19.^77^

### 4.2 Signal transducer and activator of transcription 3 (STAT3) and mitogen‐activated protein kinase (MAPK)

Furthermore, STAT3, MAPK, and phosphatidylinositol 3 kinase/Protein kinase BCD38 (PI3K/Akt) pathways together with IL‐6 and IL‐6 receptor maintain viability and sustainability of MM cell.^78^, ^79^ Similarly, heat‐shock protein 90 (Hsp90) overexpression is linked with the growth and expansion of MM cells.^78^

In Covid‐19, these inflammatory signaling are highly activated. MAPK is triggered in SARS‐CoV‐2 infection by direct activation or downregulation of ACE2.^80^ Activated MAPK in Covid‐19 causes thrombosis, inflammation, and vasoconstriction.^80^ Similarly, the STAT3 pathway, which is involved in the pathogenesis of SARS‐CoV‐2 infection, is stimulated in Covid‐19 by proinflammatory cytokines.^81^ The activated STAT3 pathway promotes thrombosis, inflammation, and lung fibrosis with impairment of viral immune response and development of lymphopenia.^81^ Therefore, STAT3 inhibitors could be potential therapy against Covid‐19 pathogenesis and cytokine storm development.^82^ Indeed, SARS‐CoV‐2 infection stimulates PI3K/Akt pathways leading to inflammation and promoting viral pathogenesis.^83^

### 4.3 Hsp90 and RANKL

Further, Hsp90 stabilizes viral proteins and promotes viral replication so that Hsp90 inhibitors could be effective against SARS‐CoV‐2 infection.^84^ Hsp90 is highly expressed in cancer cells, including MM, and is engaged with oncogenic pathways.^85^ Therefore, inhibition of Hsp90 could be a promising option in treating MM.^85^ Furthermore, Hsp90 inhibitors like NUV‐AUY922 and KW‐2478 prevent MM's chemotherapy resistance by suppressing ERK/Akt and NF‐κB signaling pathway.^86^ Therefore, Hsp90 inhibitors might be a therapeutic strategy to treat MM and Covid‐19.

The activation of RANKL in MM activates osteoclasts in bone marrow stroma, resulting in osteolytic lesions and calcium release.^16^ Tao et al. hypothesized that high proinflammatory cytokine, hypoxia, and HIF‐1 promote the expression of RANKL on monocytes with induction of osteoclastogenesis.^87^ In addition, prolonged hypoxia increases osteoclast activity by inducing pro‐osteoclastogenic factors like colony‐stimulating and VEGF.^87^

Thus, exaggerated immune response and proinflammatory cytokines may exacerbate the bone resorptive process and osteolytic lesions in patients with MM.

### 4.4 Nuclear factor kappa B (NF‐κB) and IL‐6

In Covid‐19, NF‐κB and IL‐6 are highly activated and linked with the development of cytokine storm.^88^ In SARS‐CoV‐2 infection, viral Pathogen‐associated molecular patterns (PAMPs) activate the release of IL‐6 and NF‐κB in immune and nonimmune cells, causing immune response stimulation.^88^ Furthermore, upregulation of AngII by downregulation of ACE2 in SARS‐CoV‐2 infection also triggers activation of NF‐κB and IL‐6.^89^ In addition, IL‐6 is regarded as a potent stimulator of the STAT3 signaling pathway in Covid‐19.^89^ Ultimately, activation of NF‐κB and IL‐6 in Covid‐19 propagates cytokine storm progression and MOI development.^90^

Of note, high IL‐6 concentration is linked with osteoporosis and angiogenesis in MM.^21^ Furthermore, IL‐6 from myeloid lineage, chiefly from myeloid cells, is predicted to be a proliferator factor for plasma cells in MM.^91^ A retrospective study involving 105 MM patients compared with 20 healthy controls showed that IL‐6 serum level was higher in MM patients and connected with poor clinical outcomes than healthy controls.^92^ Therefore, inhibition of IL‐6 by curcumin^93^ and metformin^21^ may reduce pathogenic properties of plasma cells in MM and could be of therapeutic value in the management of MM. both metformin and curcumin had been observed and proposed to be effective against the pathogenesis of SARS‐CoV‐2 infection.^94^, ^95^

Similarly, the NF‐κB signaling pathway is over‐exuberant and involved in the pathogenesis in MM. Mutations in the main components of the NF‐κB signaling pathway are found in 17% of MM tumors and 42% of MM cell lines.^96^ In addition, NF‐κB regulates multiple genes engaged with the proliferation of MM plasma cells.^96^ Roy and his coworkers found that the NF‐κB signaling pathway was related to the induction of drug resistance and had a role in therapeutic and prognostic outcomes.^97^ Therefore targeting this pathway may reduce the pathogenesis and severity of MM. anti‐CD38 and antiprogrammed cell death‐1 and its ligand, which inhibits the NF‐κB signaling pathway, successfully managed MM.^98^, ^99^

Since NF‐κB/IL‐6 signaling axis is activated in Covid‐19 and appoints pathogenesis and severity of MM, targeting this axis may reduce the severity of both Covid‐19 and MM. NF‐κB/IL‐6 inhibitors may reduce the impact of SARS‐CoV‐2 infection on the disease severity in patients with MM.

### 4.5 RAGE and high mobility group box‐1 (HMGB‐1)

Advanced glycation end‐products (AGEs) and their receptors AGE (RAGE) are activated in SARS‐CoV‐2 infection leading to hyperinflammation and different complications.^100^ RAGE is a proinflammatory receptor of pattern recognition receptors superfamily expressed in immune and nonimmune cells, mainly in pulmonary epithelial cells.^101^ RAGE is activated by different ligands, including AGEs, HMGB‐1, S100 protein, lysophosphatidic acid (LPA), macrophage 1 antigen (Mac‐1), and amyloid β peptide (AβP).^102^ RAGE complex activates MAPK and NF‐κB with subsequent release of proinflammatory cytokines in SARS‐CoV‐2 infection.^100^ Soluble RAGE (sRAGE) is protective while membrane RAGE (mRAGE) is harmful in different inflammatory diseases, and Covid‐19 is linked with the development of ALI and ARDS.^100^ Thus, RAGE inhibitors might be a novel treatment target for preventing and reducing the progression of Covid‐19. RAGE inhibitors like azeliragon and TTP488 prevent RAGE from interacting with many ligands, including AGEs, HMGB‐1, S100 protein, LPA, Mac‐1, and AβP, which are involved in the pathogenesis of SARS‐CoV‐2 infection, limiting pulmonary inflammation, and ALI/ARDS that are observed in Covid‐19.^103^, ^104^

Furthermore, the HMGB‐1 plasma level was increased in patients with severe Covid‐19 and correlated with inflammatory biomarkers and intensive care unit mortality after adjusting for confounding factors.^105^ HMGB‐1 is regarded as DAMPs and can trigger inflammation in SARS‐CoV‐2 infection through activation of TLR4 and RAGE, leading to the release of proinflammatory cytokines and the development of cytokine storm.^105^ HMGB‐1 inhibitors like glycyrrhizin, hydroxychloroquine, and FPS‐ZM1can reduce Covid‐19 severity.^106^

Herein, inhibition of RAGE/HMGB‐1 1 axis can decrease SARS‐CoV‐2 infection pathogenesis and Covid‐19 severity.

On the other hand, RAGE/HMGB‐1 axis is involved in the pathogenesis of MM. Guo et al. revealed that HMGB‐1 was linked with angiogenesis, inflammation, DNA damage, and malignant proliferation of plasma cells in MM.^107^ HMGB‐1 augments the expression of antiapoptotic proteins, which can protect malignant cells from apoptosis.^107^ Besides, HMGB‐1 increases drug resistance in MM, so targeting HMGB‐1 in MM may improve chemotherapy sensitivity.^108^ Upadhyay et al. found that light‐chain‐mediated proximal renal injury was mediated by induction of HMGB‐1.^109^ HMGB‐1 inhibitors like metformin^110^ may attenuate HMGB‐1‐induced complications in patients with MM.

Furthermore, the AGEs/RAGE axis is upregulated due to oxidative stress in cancer, leading to tumor progression by activating p53, HIF‐1, and nuclear erythroid‐related factor‐2.^111^, ^112^ In a prospective study comprising 19 MM patients compared with 16 healthy controls, AGE plasma level was increased while sRAGE was reduced in MM patients compared to the controls.^111^ Thus, activation of sRAGE and inhibition of the mRAGE/RAGE axis might be of value in the management of MM.

These observations suggest that RAGE/HMGB‐1 1 axis is interrelated in the pathogenesis of SARS‐CoV‐2 infection and MM, and targeting this axis may attenuate the development of Covid‐19‐related complications in patients with MM.

### 4.6 Autophagy and mechanistic target of the rapamycin (mTOR) pathway

The autophagy and mTOR pathway is connected with the pathogenesis of SARS‐CoV‐2 infection. The interplay between autophagy and SARS‐CoV‐2 is not entirely understood since this virus, and other coronaviruses can activate and inhibit the process of autophagy, with interaction at different levels with apoptosis and Interferons response.^113^ As part of the immune defense mechanism, autophagy/macroautophagy targets viral components for lysosomal degradation and initiates exposure and expression of PAMPs to enhance viral recognition.^114^ However, SARS‐CoV‐2 can evade and evolve complex strategies to block the autophagy process and even increase its replication. SARS‐CoV‐2 proteins like open reading frame (ORF3a) E, M, and ORF7a can induce autophagosome accumulation, while SARS‐CoV‐2 protein Nonstructural protein 15 prevents autophagosome formation.^114^ The overall effect of SARS‐CoV‐2 is inhibition of the autophagic process by inhibiting fusion between lysosomes and autophagosomes with reduced lysosomal acidity.

Similarly, SARS‐CoV‐2 infection activates the mTOR pathway by blocking autophagy leading to an increased release of proinflammatory cytokines, including IL‐6, with the development of cytokine storm.^115^ Thus, mTOR inhibitors might also act as an immune regulator of cytokine storm via modulation of release of IL‐6.^115^ Therefore, these verdicts indicated that inhibition of autophagy and induction of the mTOR pathway in SARS‐CoV‐2 infection might increase Covid‐19 severity.

In MM, the autophagy process promotes the viability of MM cells. Therefore, the use of autophagy inhibitors might enhance the cytotoxic response of chemotherapy against MM.^116^ Thus, the autophagy process is a prosurvival mechanism needed for drug resistance in MMH. Hence, autophagy inhibitors and anti‐MM could enforce and increase the effect against resistance MM‐plasma cells and make autophagy a new potential therapeutic target.^116^ Recently, it has been shown that the 5‐α reductase enzyme promotes autophagy in MM, and 5‐α reductase inhibitor dutasteride could effectively manage MM by inhibiting autophagy.^117^

Besides, the mTOR pathway is activated in MM and promotes the survival of malignant plasma cells within the bone marrow, increasing resistance capacity against cytotoxic therapy.^118^ Thus, inhibition of this central pathological pathway could be a promising therapeutic target in managing MM.^118^ Ma et al. observed that resveratrol had anti‐MM effects by regulating autophagy and apoptosis by inhibiting the mTOR pathway and activating adenosine monophosphate protein kinase.^119^

These findings pointed out that the mTOR pathway and autophagy are dysregulated in both SARS‐CoV‐2 infection and MM; these pathways could link MM severity in Covid‐19.

### 4.7 CD147

In Covid‐19, SARS‐CoV‐2 exploits CD147 as an entry point for host cells.^120^ CD147 has also known as basigin or Extracellular matrix metalloproteinase inducer a putative receptor for SARS‐CoV‐2.^120^ Expression of CD147 in the endothelial cell is increased with advanced age. As well, CD147 expression is augmented in various cardiometabolic disorders, including obesity, hypertension, diabetes, thrombosis, and chronic kidney diseases.^121^ A high level of CD147 in the plasma of obese patients is correlated with vascular dysfunction and linked with cardiometabolic risk factors.^121^ This might explain high mortality and Covid‐19 severity in old age with comorbidities. A cohort study involving 119 Covid‐19 patients compared to 69 controls illustrated that platelet expression of CD147 was increased, causing inflammation and thrombosis through induction release of P selectin and HMGB1, which predict worse outcomes and Covid‐19 severity.^122^ Therefore, inhibition of CD147 by azithromycin and melatonin can decrease the severity of SARS‐CoV‐2 and Covid‐19‐related complications.^123^, ^124^

Remarkably, CD147 expression is linked with the progression of MM and other malignant cells through induction expression of sodium−proton exchangers, which increase the glycolytic pathway.^125^ Zhu et al. revealed that the cyclophilin A‐CD147 complex promotes the proliferation and homing of MM cells.^126^ A case‐control study involved 62 newly diagnosed MM patients compared with 25 healthy control subjects. Illustrated soluble (sCD147) serum level was increased in MM patients and associated with poor prognosis.^127^ Therefore, anti‐CD147 or anticyclophilin inhibits the proliferation of MM cell lines.^128^

Hence, from these findings, CD147 seems to be a potential link between MM and Covid‐19, and the use of anti‐CD147 agents might be of value in treating both MM and Covid‐19.

**Figure 2:**
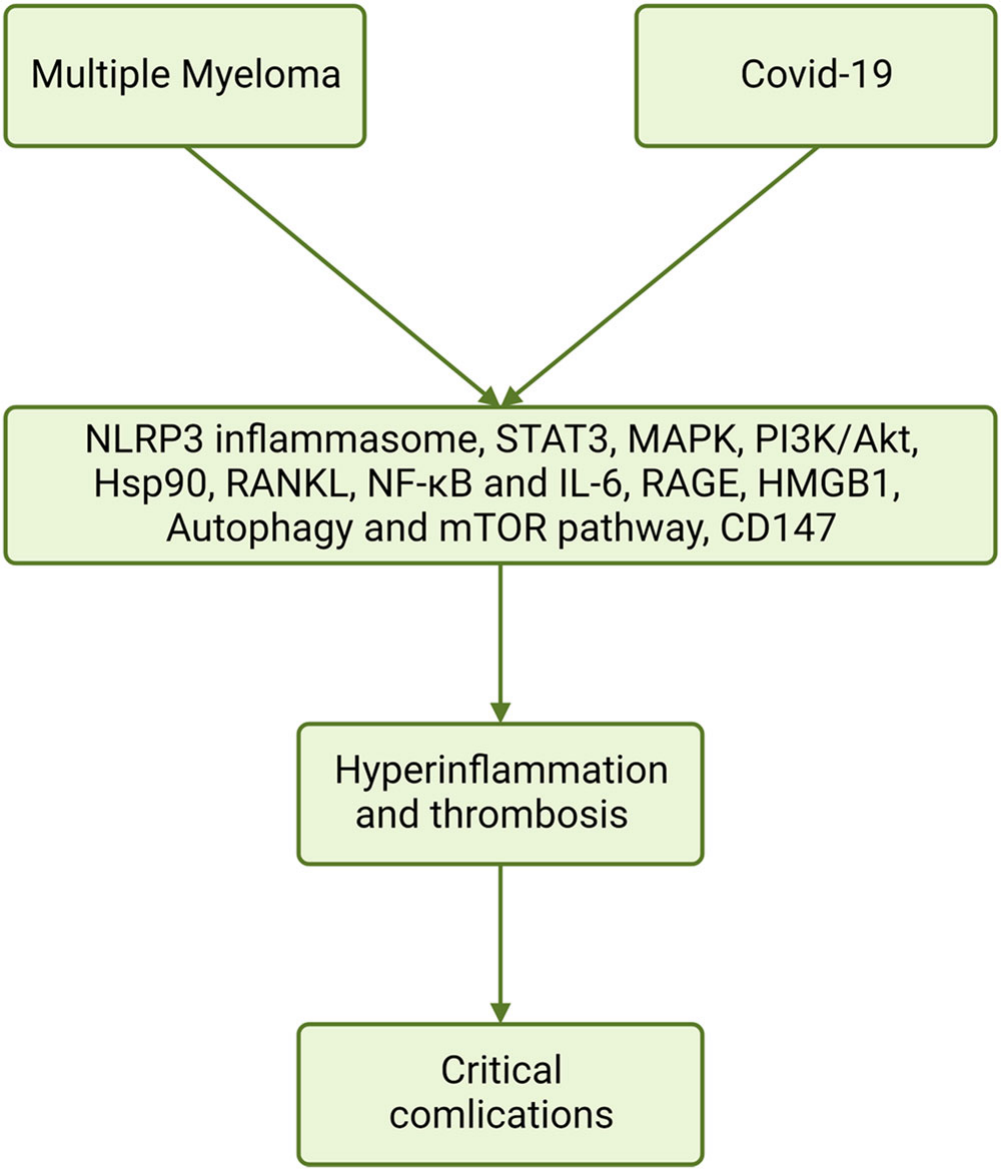
Activation of inflammatory signaling pathways in Covid‐19 and multiple myeloma: Nod‐like receptor pyrin 3 inflammasome, signal transducer and activator of transcription 3 (STAT3), mitogen‐activated protein kinase (MAPK), and phosphatidylinositol 3 kinase/Protein kinase BCD38, heat shock protein 90 (Hsp90), receptor activator of nuclear factor kappa‐Β (NF‐κB) ligand (RANKL), NF‐κB, IL‐6, Advanced glycation end‐products (AGEs), high mobility group box‐1 (HMGB‐1), mechanistic target of the rapamycin (mTOR), CD147.

These verdicts suggest that many inflammatory signaling pathways are activated in both MM Covid‐19, causing hyperinflammation, thrombosis, ALI/ARDS, and fatal complications (Figure 2).

## 5 COVID‐19 IN PATIENTS WITH MM

It has been shown in a retrospective study that severe inflammatory and immunological responses in SARS‐CoV‐2 infection in patients with MM were associated with high mortality due to hypogammaglobinemia and immunosuppression.^129^ However, immune paresis and therapy of MM did not affect the outcomes compared to the general population.^129^ However, immunosuppression in MM patients MIGHT not be the critical factor affecting the course of SARS‐CoV‐2 infection. Despite cellular and humoral immune dysfunction in MM patients, the immune response against SARS‐CoV‐2 infection was robust.^130^ Therefore, for MM patients, maintenance therapy should be continued during the development of Covid‐19, while autologous stem cell transplant recommends postponing after Covid‐19 recovery in MM patients.^131^ Furthermore, MM frequently affects elderly patients due to immunosenescence and comorbidities, which are the potential risk factor for Covid‐19.^132^ Indeed, younger patients with MM are usually treated by a high dose of chemotherapy and stem cell transplant.^132^

Therefore, MM patients are highly susceptible to Covid‐19 severity and poor clinical outcomes.^132^ Malard et al. revealed that the management of MM patients is possibly challenged during the Covid‐19 pandemic despite the use of similar guidelines.^133^ A large‐cohort study from New York City in 2020 found that the case fatality rate was 29% in MM patients with Covid‐19.^134^ A prospective study revealed that MM patients were vulnerable to adverse Covid‐19 outcomes under some immunomodulatory treatments. Besides, MM patients under daratumumab and/or lenalidomide treatments are at higher risk for Covid‐19 severity and mortality that need close follow‐up.^135^

Into the bargain, MM patients had an impaired response to the mRNA Covid‐19 vaccine than those vaccinated with BNT162b2.^136^ Of note, 55% of MM patients did not fully respond to different types of Covid‐19 vaccination due to underlying immunosuppression and cytotoxic chemotherapy.^136^ In addition, MM patients have a suboptimal immune response after SARS‐CoV‐2 vaccination and a lower seroconversion rate after Covid‐19.^137^ Therefore, booster doses are recommended in MM patients without previous SARS‐CoV‐2 infection. Stampfer et al. reported severe breakthrough SARS‐CoV‐2 infection 10 weeks following Covid‐19 vaccination in patients with variant MM.^138^

These findings highlighted that MM patients might be a risk group for Covid‐19 severity due to underlying immunosuppression and most of those patients need specific management in the Covid‐19 era.

However, the present critical review proposed various mechanistic pathways through which SARS‐CoV‐2 infection may precipitate clinical outcomes and complications in MM patients. Findings of the present review exciting further preclinical and clinical studies to confirm the robust link between Covid‐19 and MM.

The present study had several limitations, including a paucity of clinical studies linking the association between Covid‐19 and MM concerning signaling pathways. This critical review proposed that SARS‐CoV‐2 infection may increase complications in MM patients by inducing inflammatory signaling pathways involved in the pathogenesis of Covid‐19. Targeting these inflammatory pathways could be a promising therapeutic modality in managing MM patients with Covid‐19.

## 6 CONCLUSIONS

Covid‐19 is considered a primary respiratory disease‐causing pneumonia, leading to ALI and ARDS in severe cases. Covid‐19 may coexist with MM. In addition, ARDS may develop in both MM and Covid‐19 due to hypercalcemia and proteasome dysfunction. Furthermore, the expression of angiogenic factors and glutamine deficiency could link Covid‐19 severity and MM in the pathogenesis of cardiovascular and thrombotic complications. Moreover, Covid‐19 may exacerbate the development of acute kidney injury and neurological complications in MM patients. These findings highlighted that MM patients might be a risk group for Covid‐19 severity due to underlying immunosuppression and most of those patients need specific management in the Covid‐19 era. SARS‐CoV‐2 infection may increase complications in MM patients by inducing inflammatory signaling pathways involved in the pathogenesis of Covid‐19. Targeting these inflammatory pathways could be a promising therapeutic modality in managing MM patients with Covid‐19. However, the present critical review proposed various mechanistic pathways through which SARS‐CoV‐2 infection may precipitate clinical outcomes and complications in MM patients. Further preclinical and clinical studies are warranted to confirm the robust link between Covid‐19 and MM.

## AUTHOR CONTRIBUTIONS

*Conceptualization, methodology and supervision*: Hayder M. Al‐Kuraishy and Gaber El‐Saber Batiha. *Writing the original draft and collecting data*: Hayder M. Al‐Kuraishy, Ali I. Al‐Gareeb, and Ali A Mohammed. *Critical comments on the final manuscript, review, and editing*: Athanasios Alexiou, Marios Papadakis, and Gaber El‐Saber Batiha. All the authors read and approved the final version of the manuscript.

## CONFLICT OF INTEREST

The authors declare no conflict of interest.

## Data Availability

All the data are included in the manuscript. There is no new data generated.
