# Correction to “The potential link between Covid‐19 and multiple myeloma: A new saga”

**DOI:** 10.1002/iid3.1283

**Published:** 2025-06-20

Al‐Kuraishy HM, Al‐Gareeb AI, Mohammed AA, Alexiou A, Papadakis M, Batiha G‐S. The potential link between Covid‐19 and multiple myeloma: a new saga. *Immun Inflamm Dis*. 2022;10:e701. doi:10.1002/iid3.701

In the 2. MM Section, the sentence “MM is more common in men around the age of 60 years, with a prevalence of 0.7%. Without treatment, the median survival of patients with MM is approximately 7 months. Treatment survival is 4−5 years, and 5‐year survival is 54%.^14^” is incorrect. The sentence should have read: “Multiple myeloma occurs more frequently in men compared to women, affecting an estimated 230,000 individuals globally over a 5‐year period. Typically, patients are diagnosed between the ages of 66 and 70. However, about 37% of patients are under 65 years old. Advances in treatments have progressively improved the 5‐ and 10‐year survival rates, allowing those with multiple myeloma to live longer.^14,139,140^,” where reference 139 is Zhou L, Yu Q, Wei G, et al. Measuring the global, regional, and national burden of multiple myeloma from 1990 to 2019. *BMC Cancer*. 2021;21:606. https://doi.org/10.1186/s12885-021-08280-y and reference 140 is Ludwig H, Novis Durie S, Meckl A, Hinke A, Durie B. Multiple myeloma incidence and mortality around the globe; interrelations between health access and quality, economic resources, and patient empowerment. *Oncologist*. 2020;25(9):e1406‐e1413. https://doi.org/10.1634/theoncologist.2020-0141.

We apologize for this error.

